# The biology of malignant breast tumors has an impact on the presentation in ultrasound: an analysis of 315 cases

**DOI:** 10.1186/1472-6874-13-47

**Published:** 2013-11-19

**Authors:** S Wojcinski, N Stefanidou, P Hillemanns, F Degenhardt

**Affiliations:** 1 Department for Obstetrics and Gynecology, Hannover Medical School, Hannover, Germany; 2 Department for Obstetrics and Gynecology, Helios Hospital Krefeld, Krefeld, Germany; 3 Department for Obstetrics and Gynecology, Franziskus Hospital Bielefeld, Bielefeld, Germany

**Keywords:** Breast ultrasound, Cancer detection, Ultrasound features, Tumor biology

## Abstract

**Background:**

The aim of this study was to evaluate the relation of some ultrasound morphological parameters to biological characteristics in breast carcinoma.

**Methods:**

Ultrasound data from 315 breast masses were collected. We analyzed the ultrasound features of the tumors according to the ACR BI-RADS®-US classification system stratified by hormone receptor status, HER2 status, histology grade, tumor type (ductal versus lobular), triple-negativity, breast density, tumor size, lymph node involvement and patient’s age.

**Results:**

We found a variety of ultrasound features that varied between the groups. Invasive lobular tumors were more likely to have an angulated margin (39% versus 22%, p = 0.040) and less likely to show posterior acoustic enhancement (3% versus 16%, p = 0.023) compared to invasive ductal carcinoma. G3 tumors were linked to a higher chance of posterior acoustic enhancement and less shadowing and the margin of G3 tumors was more often described as lobulated or microlobulated compared to G1/G2 tumors (67% versus 46%, p = 0.001). Tumors with an over-expression of HER2 exhibited a higher rate of architectural distortions in the surrounding tissue, but there were no differences regarding the other features. Hormone receptor negative tumors were more likely to exhibit a lobulated or microlobulated margin (67% versus 50%, p = 0.037) and less likely to have an echogenic halo (39% versus 64%, p = 0.001). Furthermore, the posterior acoustic feature was more often described as enhancement (33% versus 13%, p = 0.001) and less often as shadowing (20% versus 47%, p < 0.001) compared to hormone receptor positive tumors.

**Conclusion:**

Depending on their biological and clinical profile, breast cancers are more or less likely to exhibit the typical criteria for malignancy in ultrasound. Moreover, certain types of breast cancer tend to possess criteria that are usually associated with benign masses. False-negative diagnosis may result in serious consequences for the patient. For the sonographer it is essential to be well aware of potential variations in the ultrasound morphology of breast tumors, as described in this paper.

## Background

Breast cancer is not a single disease. A great diversity concerning histopathology, immunohistochemistry, genetics and clinical presentation must be considered. The knowledge about fundamental tumor characteristics is gradually evolving and the elementary pathological division of breast tumors into ductal, lobular and other types becomes more and more complex.

The Nottingham modification of the Bloom-Richardson grading system, also known as Nottingham Histology Grade (NHG), provides a mean for the description of tumor biology [1,2]. Low-grade tumors (i.e. G1) imply a better prognosis than high-grade tumors (i.e. G3) [3]. In 1960, Elwood Jensen first described the estrogen receptor (ER) and provided the basis for a more profound understanding of breast cancer [4]. Over-expression of the human epidermal growth factor receptor 2 (HER2) in breast cancer was recognized in the 1980s and provided both a prognostic factor and a predictive factor [5]. HER2-overexpressed tumors are known to progress rapidly and have a short interval to distant metastases [6]. On the other hand, the HER2-receptor is the target for an effective therapy, the antibody-based drug Trastuzumab [7].

Nowadays, a much deeper insight into the molecular backgrounds of breast cancer exists. Just recently, gene expression profiles demonstrated that there are at least five different intrinsic subtypes of breast cancer (luminal A, luminal B, claudin-low, HER2-enriched, and basal-like) [8-10]. The progression of a tumor, the time and the pattern of distant metastasis and finally the prognosis of the disease are all highly driven by factors that are intrinsic to the distinct tumor type. The tumor biology may also have an influence on the presentation of malignant lesions in breast imaging.

Breast ultrasound, alone or as an adjunct to mammography, is a precise imaging modality with high sensitivity and specificity in the evaluation of breast lesions [11-13]. The standardized American College of Radiology BI-RADS®-US-classification for breast tumors provides a variety of categories with predefined terminology to describe the sonographic appearance of a breast lesion [14].

The accurate prediction of the malignant or benign character of a lesion plays a crucial role for the patient, but false-negative and false-positive results may occur. Breast cancer may simulate a benign lesion and vice versa, as there is some overlap in the sonographic features of malignant and benign tumors. Triple-negative breast cancer (TNBC), for example, exhibits significantly different features in ultrasound than non-TNBC [15]. False-positive diagnosis may result in an elevated rate of unnecessary biopsies. The false-negative diagnosis of breast cancers may result in delayed diagnosis and a worse outcome for the patient. Therefore, knowledge about the classic presentation of breast cancer in ultrasound and possible variations in distinct subtypes of breast cancer is crucial for the examiner to determine the malignant or benign character of a lesion precisely.

We scrutinized whether the sonomorphology of malignant breast tumors is correlated to biological features of the tumor.

## Methods

### General design and image database

Our study was carried out at the Breast Cancer Center of Franziskus Hospital in Bielefeld, Germany. Patients with a sonographically visible lesion that proved to be malignant were regarded as being suitable for our study. Patients with recurrent breast cancer, inflammatory breast cancer and tumors involving the skin were excluded.

From the hospital database, 435 consecutive breast cancer patients who attended our institution between October 2008 and January 2011 were retrospectively collected. Digitally recorded ultrasound images were available for 383 of the 435 breast cancer patients. Of these patients, 62 were excluded as they presented non-invasive breast cancer (ductal carcinoma in situ, DCIS) and 6 were excluded as data concerning medical history, receptor status, tumor stage, and/or treatment were missing. Following their exclusion, we created a database containing clinical data and digital ultrasound images from 315 patients.

As the ultrasound images had been obtained using a standard of care clinical protocol within the routine practice of our breast cancer center, our institutional ethics committee did not require additional approval for this non-interventional retrospective study design. The underlying ultrasound examinations were performed by one of four senior consultants in breast diagnostics, all of whom had at least 5-years’ experience in breast ultrasound. The examiners applied two high-end ultrasound scanners: The Siemens ACUSON S2000™ ultrasound system (Siemens Medical Solutions, Inc, Mountain View, CA, USA) equipped with the 18 L6 HD linear transducer (5.5–18 MHz, 5.6 cm) and the Hitachi HI VISION 900 ultrasound system (Hitachi Medical Corporation, Inc, Tokyo, Japan) equipped with the EUP L54M linear transducer (6–13 MHz, 5.0 cm). As standard of care, all patients received bilateral whole breast ultrasound and sonographic evaluation of the axillary regions. According to the diagnostic standards, the B-mode pictures of the tumor were documented in two planes (sagittal and horizontal).

### Image analysis

The anonymized image database was analyzed by the author SW, a DEGUM (German Society for Ultrasound in Medicine) level II certified senior consultant in gynecology with 7 years’ experience in breast ultrasound [16]. SW was blinded to the patients’ characteristics and histological results and evaluated the 315 lesions according to the ACR BI-RADS®-US classification system and the recommendations of the DEGUM [14,17]:

•Shape: Oval, round or irregular;

•Orientation: Horizontal (i.e. parallel), indifferent (including round), vertical or not determinable;

•Margin: Circumscribed or not circumscribed (with any of the following)

•Indistinct margin: Yes or no;

•Lobulated margin: Yes or no;

•Microlobulated margin: Yes or no;

•Angulated: Yes or no;

•Spiculated: Yes or no;

•Lesion boundary: Echogenic halo or abrupt interface;

•Echo pattern: Anechoic, hypoechoic, isoechoic, hyperechoic or complex;

•Posterior acoustic features: Shadowing, no posterior acoustic features, enhancement or combined pattern;

•Architectural distortion of the surrounding tissue: Yes or no;

•Changes in Cooper’s ligaments: Disrupted or displaced.

Then, the results from the systematic image interpretation were merged with the clinical data of the patients. Comparisons of baseline demographic data, tumor characteristics, and ultrasound features were made between the following groups:

•Breast density: ACR 1 and 2 versus ACR 3 and 4;

•Tumor type: invasive ductal carcinoma (IDC) versus invasive lobular carcinoma (ILC);

•Tumor grade: G1 and G2 versus G3 ; G1 versus G2 and G3;

•HER2 status: negative versus positive;

•Hormone receptor (HR) status: negative versus positive;

•Lymph node involvement: N0 versus N+;

•Tumor size: T1 versus T2, T3 and T4;

•Age: <40 years versus >40 years; <50 years versus >50 years; <60 years versus >60 years.

•Triple-negativity: TNBC versus non-TNBC.

Concerning the tumor type, most of the cases revealed to be IDC or ILC (84.7%). Therefore, other rare types (e.g. mucinous, medullary, tubular, mixed forms) were excluded from this distinct analysis. Vascularity and elasticity were not analyzed, as there were not enough images in our database that displayed these features.

### Pathology and immunohistochemistry

All pathological and immunohistochemical examinations concerning the workup of the tumor tissue were routinely performed by the pathology lab of our breast cancer center. The laboratory regularly participates in the recommended round robin tests for quality assurance. ER, PR, and HER2 were determined by immunohistochemistry. For ER and PR, the cutoff level for receptor positivity was defined as ≥1%. HER2 positivity was defined as strong complete membrane staining of ≥ 10%of the tumor cells (i.e. Score 3+). An additional fluorescent *in situ* hybridization (FISH) assay was performed to detect possible gene amplification and HER2 positivity for Score 2+. Score 1+ and Score 0 were defined as HER2-negative.

### Statistical analysis

We collected our data using Microsoft® Office Excel® 2007 (Microsoft Corporation). The author NS performed the statistical analysis and the results were validated by the author SW. The analysis was performed using MedCalc® 11.6 statistical software (MedCalc Software bvba, Belgium). The Student’s t-test was used for numerical data and comparison of means. Ultrasonographic features of TNBC and non-TNBC were compared using Fisher’s exact test for univariate distributions and Yates’ chi-square test for multivariate distributions of categorical data. When Yates’ chi-square test was found to be significant, pairwise comparisons were performed using Fisher’s exact test. Statistical significance was assumed at p < 0.05 for all tests.

## Results

**Figure 1:**
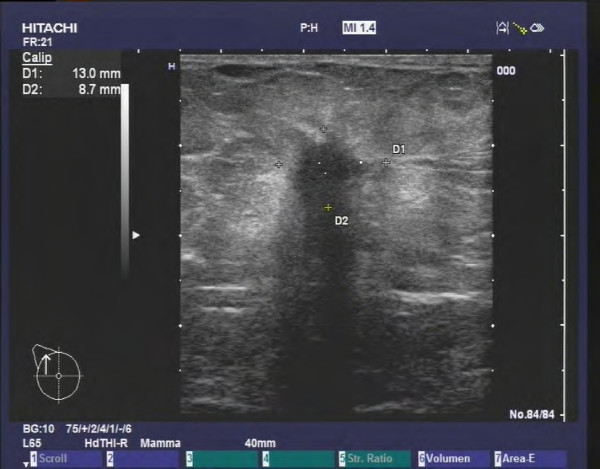
**Invasive ductal carcinoma in a 72 year old patient (HR positive, Her2 negative, G2).** The tumor exhibits typical ultrasound criteria for malignancy (irregular, hypoechoic mass with an indistinct, spiculated margin, an echogenic halo, posterior shadowing and architectural distortion of the surrounding tissue).

**Figure 2:**
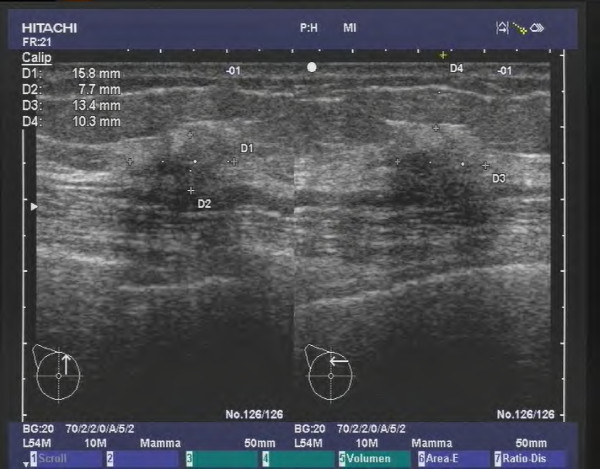
**Invasive lobular carcinoma in a 75 year old patient (HR positive, Her2 negative, G2).** The tumor appears as a hypoechoic architectural distortion with an indistinct, angulated margin.

**Figure 3:**
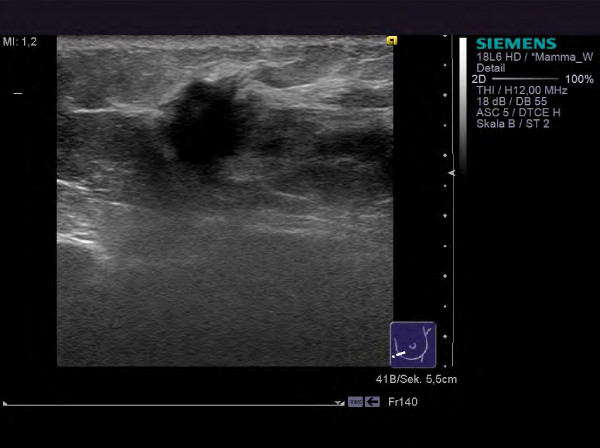
**Her2/neu-positive HR-positive tumor in a 57 year old patient (G3, invasive ductal).** The tumor presents as a hypoechoic mass with relevant architectural distortion of the surrounding tissue.

**Figure 4:**
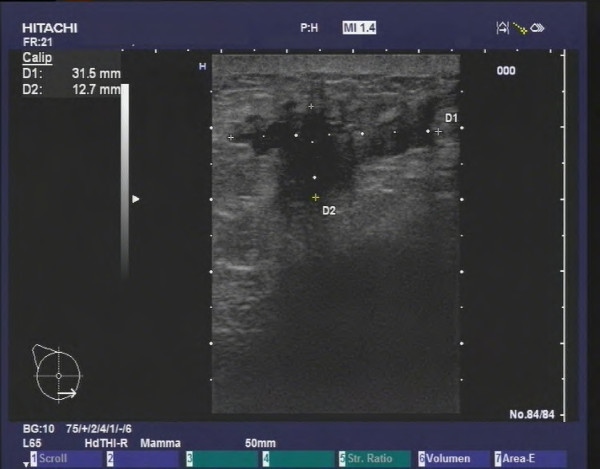
**Her2/neu-positive HR-negative tumor in a 47 year old patient (G3, invasive ductal).** The tumor presents as a bizarre, hypoechoic mass with architectural distortion of the surrounding tissue and a widely lobulated or microlobulated margin, but no echoic halo.

**Figure 5:**
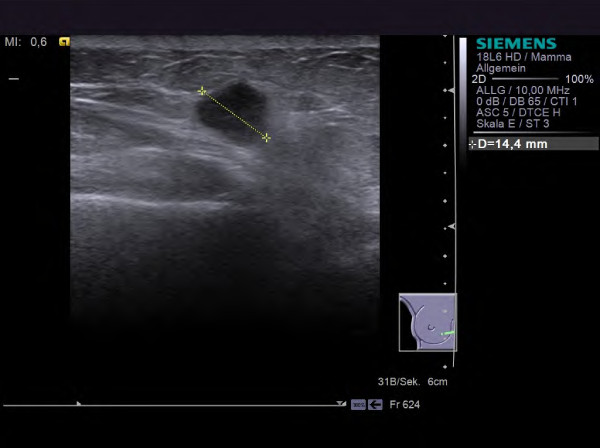
**Triple-negative breast cancer in a 52 year old patient (G3, invasive ductal).** The tumor appears as a lobulated, hypoechoic mass. The ligaments are displaced rather than disrupted.

**Figure 6:**
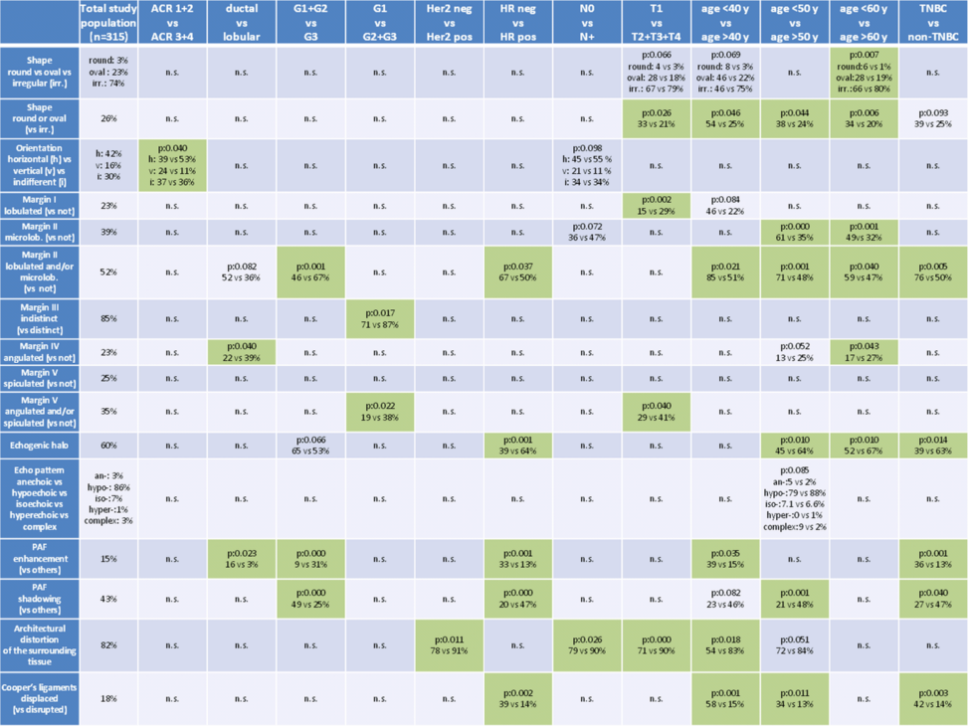
**Overview of the results I.** Influence of the tumor biology and patient’s characteristics on the sonomorphology. Significance is indicated in green. P-values greater than p = 0.100 are indicated as “not significant”. (irr. = irregular; n.s. = not significant; *TNBC* = triple negative breast cancer; *HR* = hormone receptors; *h* = horizontal; *v* = vertical; i = indifferent; microlob. = microlobulated; *PAF* = posterior acoustic features).

**Figure 7:**
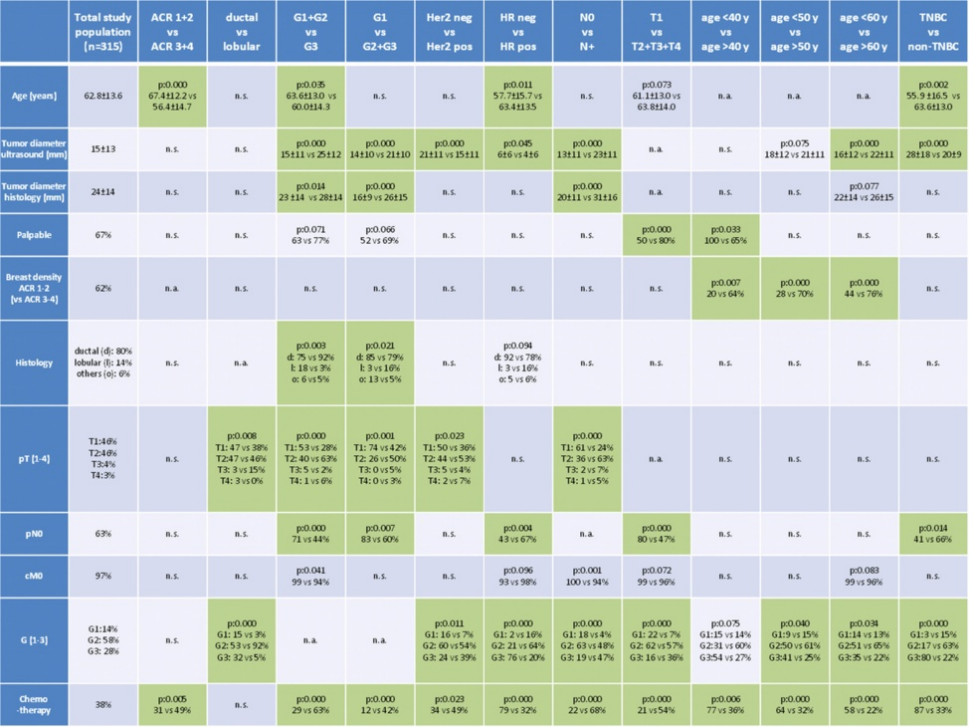
**Overview of the results II.** Influence of the tumor biology and patient’s characteristics on clinical features of the tumor. Significance is indicated in green. P-values greater than p = 0.100 are indicated as “not significant”. (*n.a*. = not applicable; *n.s*. = not significant; *TNBC* = triple negative breast cancer; *HR* = hormone receptors; d = ductal; l = lobular; o = others).

Clinical examples for different tumor types are given in Figures 1, 2, 3, 4 and 5. The results concerning the sonographic presentation of the tumors are summarized in Figure 6. The clinical aspects of the tumors are summarized Figure 7. Key aspects are described in the following paragraphs and will be discussed in the next section.

### Breast density

The pre-existing breast density had little effect on the sonomorphology of the tumors. Nevertheless, in dense breast tissue (according to the American College of Radiology, ACR 3 and 4) tumors were more likely to have a horizontal orientation than in less dense breast tissue (53% versus 39%, p = 0.04).

### Tumor type

IDC and ILC cancer showed two different ultrasound features. ILC was more likely to have an angulated margin (39% versus 22%, p = 0.040) and less likely to show posterior acoustic enhancement (3% versus 16%, p = 0.023).

### Tumor grade

Compared to moderately and well differentiated tumors, poorly differentiated tumors (i.e. G3) were linked to a higher chance of posterior acoustic enhancement and less shadowing (31% versus 9%, p < 0.001; 25% versus 49%, p < 0.001). Furthermore, the margin of G3 tumors was more often described as lobulated or microlobulated compared to G1/G2 tumors (67% versus 46%, p = 0.001).

### HER2 status

We found only one ultrasound feature that was associated with HER2-positivity. Tumors with an over-expression of HER2 exhibited a higher rate of architectural distortions in the surrounding tissue.

### Hormone receptor status

The HR status had a relevant impact on the sonomorphology. Tumors that neither expressed estrogen nor progesterone receptors were more likely to exhibit a lobulated or microlobulated margin (67% versus 50%, p = 0.037) and less likely to have an echogenic halo (39% versus 64%, p = 0.001). Furthermore, the posterior acoustic feature was more often described as enhancement (33% versus 13%, p = 0.001) and less often as shadowing (20% versus 47%, p < 0.001) compared to HR positive tumors. Finally, displacement of the Cooper’s ligaments (instead of disruption) was more often found in ER/PR negative tumors (39% versus 14%, p = 0.002).

### Tumor size

Several ultrasound features were dependent on the tumor size. Small tumors (i.e. T1) were more likely to have a round or oval shape (33% versus 21%, p = 0.026) and less likely to exhibit a lobulated or angulated/spiculated margin (15% versus 29%, p = 0.002; 29% versus 41%, p = 0.040). Furthermore, small tumors showed less architectural distortions (71% versus 90%, p < 0.001).

### Age

Patients’ age had a relevant influence on the sonomorphology of the tumors. Depending on the cut-off for the age groups, we obtained the following results: In younger patients, the tumors were more likely to present with a round or oval shape and exhibit a lobulated or microlobulated margin. On the other hand, an angulated margin was less often observed. In the group of older patients, an echogenic halo was described more frequently. In young patients, a posterior acoustic enhancement was more often seen and shadowing was less often. Furthermore, architectural distortions were less frequently described in young patients and the Cooper’s ligaments were more often described as displaced rather than disrupted.

### Triple-negative breast cancer

As described elsewhere, triple negativity of breast cancer has a relevant effect on the sonomorphology. The margin of TNBC was more frequently described as lobulated and/or microlobulated (76% versus 50%, p = 0.005) and the echogenic halo was observed significantly less often compared to non-TNBC (39% versus 63%, p = 0.014). Cooper’s ligaments were displaced rather than disrupted in TNBC in comparison to non-TNBC (42% versus 14%, p = 0.003). Posterior acoustic enhancement was more frequent in TNBC (36% versus 13%, p = 0.001) and posterior acoustic shadowing less often observed (27% versus 47%, p = 0.040).

### Tumor characteristics with impact on the sonomorphology

Overall, we performed 12 group comparisons with respect to the ultrasound features. The groups were defined either by clinical characteristics (e.g. age, breast density) or by the tumor biology (e.g. histology grade, receptor status, HER2 status). Depending on the groups observed, the number of significantly different ultrasound features varied from one different feature between groups to five different features, respectively. We found numerous differences comparing HR positive and hormone receptor negative tumors (i.e. rate of lobulated or microlobulated margin, presence of echogenic halo, posterior acoustic features and changes in Cooper’s ligaments) and comparing TNBC and non-TNBC. Furthermore, patient’s age (independent from the chosen cut-off) had a considerable effect on the sonomorphology of tumors. Regarding the tumor size, T1 tumors frequently exhibited different features than larger tumors (i.e. shape, presence of lobulated margin, rate of angulated or spiculated margin and architectural distortions) [Figure 6].

### Sonographic features that frequently varied between the groups

We compared 16 ultrasound characteristics between the groups in different categories. Focusing on the ultrasound features, the tumor biology proved a considerable effect on various elements. Amongst others, the presence of a lobulated or microlobulated margin and the presence of an echogenic halo were relevantly influenced by the tumor biology. Furthermore, the posterior acoustic features frequently varied between the investigated groups. Finally, the frequency of architectural distortions and changes of the Cooper’s ligaments were frequently dissimilar between the groups [Figure 6].

## Discussion

### Is it plausible, that the tumor biology has an impact on the sonomorphology?

Breast cancer is not merely characterized by features that can obviously be detected by clinical examination, medical imaging or visual evaluation of a tumor specimen, but rather by distinct intrinsic attributes. Essential tumor characteristics, like histology grade, hormone receptor status and HER2 expression, have a biological, proteomic or genetic background. Therefore, the characterization of tumors has moved from the macroscopic over the microscopic to the molecular dimension.

In the first instance, it has to be considered if it is plausible that differences in the molecular attributes of breast cancer can have an impact on the sonomorphology of the tumor. Ultrasound is principally capable of visualizing macroscopic qualities of a mass and thus detecting differences in the gross appearance. However, our results demonstrate that even sub-microscopic features of a tumor may modify its appearance in ultrasound. Understandably, ultrasound cannot directly detect intrinsic parameters of the tumor and it cannot be the aim to predict these parameters by imaging methods. Nevertheless, the typical ultrasound features of malignant breast masses may vary in distinct tumor types. Knowledge about these variations would help the examiner to avoid the false classification of breast lesions. Depending on the chosen groups, we detected a various number of different ultrasound features.

### Data from the literature

The common features of malignant breast tumors are described in specialized books [11]. Furthermore, the American College of Radiology (ACR) has published reference guidelines on the categorization of breast tumors according to their ultrasound characteristics [14]. However, breast cancer cannot be regarded as a single disease and according to histological, immunohistochemical or genetic features, several subtypes can be distinguished [8-10]. Although there has never been a detailed and systematic approach before, we found data in the literature that focuses on certain (sono-) morphologic features of distinct subtypes and that will be discussed in the following sections.

### Histological tumor type

ILC may be occult in both mammography and ultrasound, and breast-MRI may have certain advantages in the detection of this tumor type [18,19]. However, the sensitivity of ultrasound seems to be higher than mammography (93.9% versus 79.8%) [20]. In 2005, Watermann et al. published data on the ultrasound features of ILC cancer [21]. They found that an irregular shape, indistinct margins and posterior acoustic shadowing were described significantly more often in ILC than in other tumor types (88% versus 67%, p < 0.001; 95% versus 76%, p = 0.001; 84% versus 58%, p = 0.001, respectively). These findings partly comply with our own results (85% versus 71%, p = 0.116; 97% versus 94%, p = 0.700; 59% versus 45%, p = 0.164), as we found corresponding tendencies, but did not reach a level of statistical significance. Nevertheless, we can support the theory that histological differentiation modifies the ultrasonographic appearance of breast cancer. To our interpretation, the posterior acoustic features are of special importance in ILC. We found that posterior acoustic enhancement is observed significantly less often in ILC (3% versus 16%, p = 0.023) and either shadowing or mixed features or no features is significantly more frequently observed. The detectability of ILC is often impaired in both mammography and ultrasound as this tumor type has a diffuse and frequently multicentric growth pattern and does not present as a mass. However, a slight architectural distortion with a related posterior acoustic shadowing may be the only hint for this tumor type in ultrasound [22]. The sonographer should be aware of the distinct ultrasound features of ILC in order to avoid false-negative diagnosis. We did not focus on rare histological types of breast malignancies (e.g. mucinous, medullary, tubular, mixed forms, metastases) as these entities only represented a small number of cases in our study. However, we want to emphasize, that these tumors, in particular, tend to exhibit imaging characteristics that are unique and that may be different compared to IDC and ILC. Ultrasound features of these subtypes are described elsewhere [23-27].

### HER2 status

Focusing on the HER2 status, architectural distortions were observed significantly more often in HER2 positive tumors than in HER2 negative tumors (91% versus 78%). Gene amplification and/or protein over-expression of HER2 results in a more aggressive phenotype with increased cell proliferation, motility and tumor invasiveness, accelerated angiogenesis, and reduced apoptosis [28,29]. These biological behaviors imply a rapid infiltration and destruction of the surrounding tissue and, consequently, influence both the macroscopic growth pattern of the tumor and the appearance on ultrasound. The resulting architectural distortions are a reliable predictor for malignancy and occur in the majority of HER2 positive tumors (91%). Therefore, HER2 positive tumors may be regularly detected and classified as probably malignant by ultrasound. In the literature, we found no conclusive data that could be compared to our results.

### Hormone receptor status and triple negativity

Apparently, the HR status with the associated biological background has a strong impact on the expression of sonographic features. In an earlier analysis of 281 women, Aaltomaa et al. correlated HR status with histological variables and mitotic indices [30]. The authors described a relation to nuclear grade, tumor necrosis, tumor circumscription, inflammatory cell reaction, intraductal growth pattern and tubule formation. The authors concluded that HR negativity implies an increased proliferation rate and a number of malignant histological features in breast lesions. These histological features may explain the variation in the ultrasound characteristics between HR positive and HR negative cancers concerning a lobulated or microlobulated margin, an echogenic halo, the posterior acoustic feature and changes in the Cooper’s ligaments. Just recently, Aho et al. published data on 101 breast tumors. The authors concluded, that posterior acoustic shadowing was more often associated with ER positive tumors (90.9% versus 9.1%) and PR positive tumors (72.7% versus 27.3%). This corresponds well with our results (47% versus 20%), although the differences are less accentuated in our case series, which can be explained, as we analyzed the global HR status and did not differentiate between ER and PR [31].

### Echo pattern

The most frequently observed echo pattern in breast cancer are hypoechoic tumors (86%). We found no variables that influence the distribution of echogenicity. Our results comply with reports in the literature that also found no difference in the groups with respect to histological size, grade, axillary metastases, hormone receptor status and lymphovascular invasion [31,32].

### Patient’s age

Virtually independent from the cut-off, stratification by age revealed that younger patients were more likely to exhibit round or oval tumors and a lobulated or microlobulated margin. On the other hand, they were less likely to show an echogenic halo, architectural distortions and disruption of the Cooper’s ligaments. Consequently, the tumors may lose some of the typical criteria for malignancy in young patients and may be misjudged as benign lesions if the sonographer is not fully aware of this behavior. This phenomenon cannot be explained by the patient’s age alone, but must be considered as a coincidental effect that is mainly triggered by the distinct tumor biologies that are common in young patients. Recently, Bullier et al. published data on 97 cases of breast cancer in women under 40 years old [33]. The authors concluded, that young women have more luminal B/Her2+ phenotypes and that the appearance of cancers is correlated with their biological profiles. Consequently, sonomorphology is mainly driven by the tumor type and not by patient’s age. We support this interpretation as our results suggest the same interrelation.

### Limitations of our study

The main limitation of our study is that there was only one observer and image analysis was based on a single, previously acquired still image. Although the observer was blinded, this circumstance may impair some of the results. However, for further studies we would propose to include multiple observers and provide multiple images of each tumor or even video loops. Furthermore, the considerable number of groups and observed variables may boost statistical errors: Following stratification, we performed about 310 comparisons and calculated the statistical significance between the various groups. However, with an error rate of 5%we could expect no more than 16 positive results by chance. Finally, we found 93 differences that were statistically significant. Therefore, we consider that most of the positive results reflect objective differences between the groups. Nevertheless, it has to be considered that some of the apparent differences between groups may be invalid for statistical reasons.

## Conclusions

Precise evaluation of breast masses before further diagnostic or therapeutic steps has a crucial impact on the quality of the treatment and the outcome in the patient. Lesion assessment by ultrasound is based on the ACR BI-RADS®-US classification system that provides a substantial source for the prediction of the malignant or benign aspects of a tumor. Nevertheless, not each breast cancer follows the rules of typical ultrasound criteria for malignancy and variations may occur depending on individual factors, such as patient’s characteristics and tumor biology. Usually, round or oval shape is associated with benign lesions, but may also occur in certain types of breast cancer. An echogenic halo is an indicator for malignancy, but it is frequently absent in HR negative tumors. Moreover, posterior acoustic enhancement is associated with benign lesions, but may also occur in high-grade tumors, HR negative tumors and young patients. Finally, architectural distortions are less often observed in small tumors, young patients and Her2 negative tumors.

Therefore, false classification of breast masses may arise with all of the known consequences for the patient. For the advanced sonographer it is essential to be aware of potential variations in the ultrasound morphology of breast tumors. This knowledge would enable the examiner to improve the diagnostic accuracy in the evaluation of breast lesions and finally help to guide the patient in the proper therapeutic direction.

## Abbreviations

ACR: American College of Radiology; D: Ductal; DCIS: Ductal carcinoma *in situ*; ER: Estrogen receptor; FISH: Fluorescent *in situ* hybridization; H: Horizontal; HER2: Human epidermal growth factor receptor 2; HR: Hormone receptor; I: Indifferent; IDC: Invasive ductal carcinoma; ILC: Invasive lobular carcinoma; irr.: Irregular; l: Lobular; LN: Lymph node; Microlob: Microlobulated; n.a: not applicable; n.s: not significant; NHG: Nottingham Histology Grade; O: Others; PAF: Posterior acoustic features; PR: Progesterone receptor; TNBC: Triple-negative breast cancer; V: Vertical.

## Competing interests

The author’s declare that they have no competing interests.

## Authors’ contributions

SW contributed to the conception and design of the study and FD provided methodological advice. SW and NS performed the data collection and SW evaluated the anonymized ultrasound imaged. NS contributed to the analysis of the data and SW contributed to the interpretation of the results. SW and NS contributed to the writing of the manuscript. PH and FD conducted the final review of the data and the manuscript. SW, NS and FD were employees at the Franziskus Hospital Bielefeld at the time of the study. All authors read and approved the final manuscript.

## Pre-publication history

The pre-publication history for this paper can be accessed here:

http://www.biomedcentral.com/1472-6874/13/47/prepub
